# Dynamics and control of separable coupled rigid body systems

**DOI:** 10.1186/s40638-017-0068-0

**Published:** 2017-11-10

**Authors:** Kinda Khalaf, Dongming Gan, Hooshang Hemami

**Affiliations:** 1 Department of Biomedical Engineering, Khalifa University of Science and Technology, Abu Dhabi, UAE; 2 Robotics Institute/Mechanical Engineering Department, Khalifa University of Science and Technology, Abu Dhabi, UAE; 3 Department of Electrical and Computer Engineering, The Ohio State University, Columbus, OH 43210 USA

**Keywords:** Coupled mechanical systems, Constrained dynamics, Kinematics, Control, Dimensionality reduction

## Abstract

This paper explores the dynamics of separable coupled rigid body systems, a special class of constrained rigid body systems. These are defined as two systems that interact with each other by forces of contact, resulting in a reduction in dimensionality and complexity. The mechanics and consequences of this reduction are investigated here. The basic hypothesis and an example of the reduction in two successive steps are formulated. A simple mechanical biped model is developed and analyzed in some details by both system theoretical concepts and simulations. The main contribution of this work is the novel extension to the known dynamics of constrained rigid bodies. The modular, versatile and systematic formulation presented here is computationally efficient and has many applications in the studies of the human neuro-musculoskeletal system, robotic systems and humanoids, as well as clinical and sports biomechanics applications. Computer simulations are provided to demonstrate the feasibility and effectiveness of the methodology.

## Background

Numerous works on constrained rigid bodies and multibody dynamics (MBD) have been done over the last few decades, including several well-known textbooks [1, 2]. Various algorithms were also developed in a wide variety of fields and application areas [3–7]. Technological advancement warranted the advent of improved formulations with better computational efficiency [8]. Recent approaches such as the “divide-and-conquer” algorithm (DCA) were used to reduce the computational burden associated with many aspects of modeling, designing and simulating articulated multibody systems [7].

On the other hand, much less attention has been given to separable coupled rigid body mechanics, a particular class of constrained rigid bodies, as evidenced by the paucity of the literature in this area. Defined as systems where the physical coupling or interaction takes place primarily through the forces of contact [9], no gripping or holding occurs in such systems. In general, the two systems get separated when the forces of contact go to zero. It is well known that some human contact with certain machines or inanimate objects fall in this category. Specific examples include medical and sport-related interactions, such as rotating medical platforms with a human on the platform and humans on diving, skating or surfboards. This work extends the current body of the literature on constrained rigid body dynamics by proposing a computationally efficient formulation for separable coupled systems. We address the rigid body dynamics, stability and control of separable rigid bodies within the scope of human or robot interaction with a subset of the above three classes of objects: medical platform, human on skateboard and human on surfboard.

While the dynamics of the three situations are somewhat similar and can be formulated along the lines discussed in this paper, the control problem and objectives are, however, different. For example, in the medical platform case, the platform is independently moved, and the objective of the human or patient on the platform is to achieve postural stability, even though the exact mechanisms of such control by the central nervous system (CNS) are not well understood. In the skatingboard situation, the human controls both subsystems—the human’s and the board’s. The board does not exert any control of its own. In the surfboard scenario, the control is distributed between natural random forces that propel the board’s translational and attitudinal motion, and the partial control the rider exerts to maintain stability, hold attitude relative to the waves for some not well-defined performance index and proceed with smooth transnational motion or achieve some acceptable mixture of all these objectives. When the two subsystems are humans, the dynamics and the control problem become more involved and complicated, and this case will not be pursued here.

Systems of connected rigid bodies with holonomic, non-holonomic and soft constraints can be adequately utilized to model robotic [10], humanoid [11] and locomotion systems [12]. The interaction of a robot with a moving platform or stable base can be represented in the equations of motion. This paper extends the previous body of work to theoretically formulate the dynamics and control of a multilink mechanical system in contact with a stationary or moving base of support. We will first formulate the general two rigid body system dynamics and apply it to simple models of platform rotation and skateboarding. We will discuss the control for some simple and specific maneuvers.

A certain amount of dimensionality and complexity reduction is achieved by replacing the dynamics of one subsystem with kinematics [13]. The equations of motion and procedures for their reduction are outlined. Stability is achieved by standard feedback technique to counteract the base movement. The biped is to maintain stance and postural stability under support platform disturbance [14]. The formulation here also allows computational studies and further quantitative assessment of sensory and processing deficits in stability and balance for both healthy and injured humans [14, 15]. With regard to postural adjustments, it is hypothesized that humans use ankle strategy, hip strategy and combinations of the two in order to maintain balance [16]. The formulation allows quantitative studies of such hypotheses and the role of certain variables such as center of gravity, center of pressure, shear and normal support forces [17, 18]. It may also be relevant to studies of vestibular deficit [19, 20].

This paper is structured as follows: The dynamics of the two-coupled separable systems is formulated in second section. Two examples, a biped model under rotational platform disturbances and a simple skateboard model, are formulated in third section. Stability and control are discussed in subsection. Simulations and comparisons are presented in fourth section. Discussions and conclusions are in fifth section followed by References and Appendices.

## Coupled separable systems

For ease of comparison and reference we adopt the same notation and variable names as in Ref. [12]. The subsystems are assumed to be planar and given in the Lagrangian formulation:

1 $$I_{1} (\varTheta )\ddot{\varTheta } + B_{1} (\varTheta ,\dot{\varTheta })\dot{\varTheta } - gG_{1} (\varTheta ) = W_{1} U_{1} + [\partial C_{1} /\partial \varTheta ]\varGamma_{1}$$

2 $$I_{2} (\varPhi )\ddot{\varPhi } + B_{2} (\varPhi ,\dot{\varPhi })\dot{\varPhi } - gG_{2} (\varPhi ) = W_{2} U_{2} + [\partial C_{2}^{'} /\partial \varPhi ]\varGamma_{2}$$

The forces Γ_1_ and Γ_2_ are the forces that couple the above two subsystems. It is assumed that these are the only constraint forces that appear in the equations above with

$$\varGamma_{1} = - \varGamma_{2}$$

The algebraic constraint equations are, in general, functions of all the degrees of freedom.

3 $$\begin{aligned} C_{1} (\varTheta ,\varPhi ) = 0 \hfill \\ C_{2} (\varTheta ,\varPhi ) = 0 \hfill \\ \end{aligned}$$

**Fig. 1:**
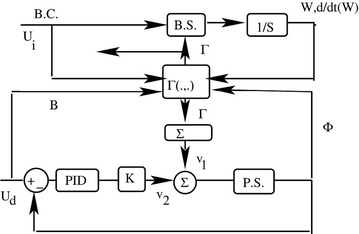
The planar one-segment biped and a particular planar platform subsystem

A block diagram of the system is shown in Fig. 1 which is described in more detail later. The system could also have been described by combining constraints and forces of constraint

4 $$\begin{aligned} & CC = \left[ {C_{1}^{\prime } ,\; - C_{2}^{\prime } } \right]^{'} \\ & \varGamma = \varGamma_{1} \\ \end{aligned}$$

The matrices *I* and *B* of the combined system are block diagonal. This latter representation is useful when one subsystem controls both subsystems. For the time being, the objective is to consider the subsystem that is being controlled, i.e., the first subsystem. This subsystem is being controlled through the forces Γ exerted by the second subsystem. Further, it is assumed that the constraints *C*_1_ can be separated into two parts as shown in Eq. (5):

5 $$C_{1} = C(\varTheta ) + D(\varPhi )$$

For the computation of the forces of constraint Γ_1_, it is necessary to differentiate equation (5) twice with respect to time. For ease of reference, the second derivative is symbolically reproduced here.

6 $$(\partial C^{\prime } /\partial \varTheta )(\ddot{\varTheta }) + \partial [(\partial C^{\prime } /\partial \varTheta )(\dot{\varTheta })]/\partial \varTheta [\dot{\varTheta }] + (\partial D^{\prime } /\partial \varPhi )(\ddot{\varPhi }) + \partial [(\partial D^{\prime } /\partial \varPhi )(\dot{\varPhi })]/\partial \varPhi [\dot{\varPhi }] = 0$$

The main point of the above equation is that the influence of the controlling subsystem on the controlled dynamics of the controlled subsystem is through Φ, $\dot{\varPhi }$ and $\ddot{\varPhi }$. In practical terms, this means that the controlled subsystem must, through its sensory apparatus, measure the forces of constraint Γ and, from these measurements and possibly its actuators dynamics that may be sensitive to the platform motion, extract the controlling subsystem’s positions, velocities and acceleration variables. This issue is further elaborated in the examples and in the control. Consider a mechanical multilinkage system with *Z* as an n-vector of the degrees of freedom, *U* as the input vector of ideal moment of force generators, Γ as the vector of constraint forces and

$$C = 0$$

as a set of holonomic constraints governing the system. The equations of motion in the Z space are:

7 $$I(Z)\ddot{Z} + B(Z,\dot{Z})\dot{Z} - gG(Z) = WU + [\partial C/\partial Z]\varGamma$$

It is well known that when the constraints are maintained, the following equations describe the behavior of the system: When the constraints are satisfied, the forces of constraint are functions of the state Z and $\dot{Z}$ and the inputs:

8 $$\begin{aligned} & C(Z) = 0 \\ & (\partial C^{{\prime }} /\partial Z)((I^{ - 1} )I\partial C/\partial Z)\varGamma = (\partial C^{{\prime }} /\partial Z)((I)^{ - 1} )( - B - gG + WU) - \partial [\partial C/\partial Z(\dot{Z})]/\partial Z(\dot{Z}) \\ \end{aligned}$$

When the constraints are not in effect,

$$\varGamma = 0$$

and

$$C \ne 0$$

Suppose the above system can be dichotomized into a dominant subspace *p*_*d*_ or simply *d* and an influenced subspace *p*_*i*_ or simply *i*. The motion of the dominant part is influenced only minimally by the motion of the influenced part. Physical examples of this situation arise, for example, when a human is on a ship, in a vehicle, on a surfingboard in water or on a moving platform. Let the lower *r* degrees of freedom of *Z* be the dominant subspace represented by vector *Y*, and an input set *U*_*d*_. Let the remaining vector *X* of *n* − *r* degrees of freedom be that of *p*_*i*_. The influence of the subspace *d* on subspace *i* is through the constraint forces that are active between the two subsystems Γ_*id*_ and the known motion *β* of the subspace *d*. The subspace *i* has its own control inputs *U*_*d*_ as part of the input vector *U*. The objective here is to arrive at the (reduced) dynamics of the subspace *i* with inputs *U*_*m*_, *β* and Γ_*id*_.

Suppose the dynamics of the subspace *d* be described by a very large moment of inertia matrix *I*_*r*_ and inputs *U*_*d*_ that are very large in magnitude. This assumption allows reduction in the dynamics of the subspace *d* to the following

9 $$I_{r} \ddot{Y} = W_{r} U_{r}$$

From the known motion *β* of the subspace *d*, the velocities $\dot{\beta }$ and the accelerations $\ddot{\beta }$ can be computed. Therefore, Eq. (9) can be replaced by

10 $$\ddot{Y} = \ddot{\beta }$$

Without loss of generality, we can assume *I*_*r*_ is a diagonal matrix of dimension *r*, and the known accelerations are a vector of dimension *r*. The dynamics of the original system of Eq. (7) can be written as that of two disjoint subspaces except that the forces of support between the two subspaces Γ_*id*_ must be computed.

The equations of motion for this combined system of dynamic subspace *i* and the kinematic subspace *d* are formulated here. Let *Z*_1_ be the vector of [*X*, *β*]. Let *I*_1_ be the two-block diagonal matrix. The upper block diagonal is the restriction of *I* to dimension *n* − *r*. The second block diagonal is the identity *rxr* matrix. The *B*_1_ matrix is the same as *B* with *Z*_1_. The same is true for *G*. The matrix *W* is the same except that all the elements in the last *r* rows are zero. Similarly, all the last *r* rows of *ӘC′/ӘZ* in Eq. (7) are set to zero. Let the latter matrix be called *V*.

However, vector *C* has remained intact and when it is differentiated with respect to time, the variables *β* remain in its last *r* columns. These last *r* columns of *C′*/*Z* do not affect the equations of motion for the combined system because the *I*_1_ matrix is block diagonal. Let us assume, for simplicity and ease of notation, that the only forces of constraint remaining in the system are Γ_*id*_. The latter restriction is not major and can easily be lifted. Let *V*_1_ be a vector comprising of all the remaining kinematic variables that have been moved to the left side of the equation from *I*, etc., namely, the kinematic degrees of freedom *β*, and their first and second derivatives with respect to time.

With these clarifications, the equations for the combined system are:

11 $$I_{1} (Z_{1} )\ddot{Z}_{1} + B(Z_{1} ,\dot{Z}_{1} )\dot{Z}_{1} - gG(Z_{1} ) = W_{1} U_{i} + V\varGamma_{id} + V_{1} (\beta ,\dot{\beta },\ddot{\beta })$$

From Eq. (11), one can derive the required forces of constraint Γ_*id*_ similar to Eq. (8). Once this last step is carried out, the original system is effectively reduced to dimension *n* − *r*.

A major step in the above development is that the computation of forces of constraint has been extended to systems described by a combined system of kinematics and dynamics. The main difference between the all dynamic and the combined kinematic–dynamic systems is that, for the latter system, the forces of constraint are functions of the kinematic accelerations, velocity and positions in addition to being functions of the state and the remaining inputs.

A more precise description of the system as described is given in Fig. 1. The upper part of the figure shows the dynamics of the biped, i.e., the *i* subsystem. The lower part of the figure shows the dynamics of a special version of a d subsystem. The two subsystems are primarily coupled through the four forces of constraint Γ. These forces are, in general, functions of the states and inputs of both subsystems. B.C. or *U*_*i*_ refers to biped controls. B.S. describes the biped dynamics. Integrators with respect to time are shown as 1/s. The variables *W* and d*W*/d*t* are, respectively, the position and velocity states of the biped. Γ is the vector of the forces of interaction between the platform and the biped. The inputs to the platform are *U*_*i*_. P.S. describes the platform dynamics. The angle of rotation of the platform is Φ. The compensator is of the PID type. The large gain is *K* and *v*_1_ describes the dynamic effect of the forces of constraint on the platform support forces acting on the system.

## Biped on a rotating platform

**Fig. 2:**
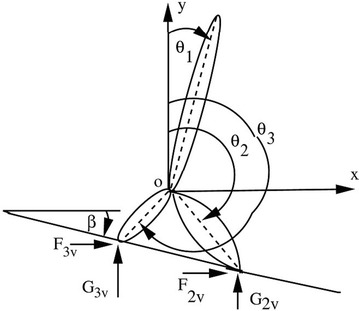
The one-segment biped with a two-segment foot and the platform support forces

As an example, consider a planar biped with a two-segment feet on a platform as shown in Fig. 2. This biped is an example of the *i* subspace. The system angles, the inertial coordinate system and the platform rotation angle: *β*—positive clockwise are shown.

The objective is to control the biped when it is disturbed by a platform rotation. The biped has five degrees of freedom: the translation of the center of gravity of the body, and the three angles of the body and the two segments of the foot relative to the vertical gravity axis. Let the angle of rotation *β* of the platform be positive in the clockwise direction—measured from the horizontal axis. Let Γ be the four forces of contact with the platform—two support forces and two shear forces

$$\varGamma = \left[ {F_{2v} ,G_{2v} ,F_{3v} ,G_{3v} } \right]^{{\prime }}$$

where the superscript means transpose.

### Biped dynamics

The equations of motion in the *W* space are

12 $$J(W)\ddot{W} + B(W,\dot{W})\dot{W} - gG(W) = EU + [\partial C/\partial W]\varGamma$$

The matrices and vectors appearing in Eq. (12) are given below.

13 $$\begin{aligned} J(\omega ) = \hfill \\ \left[ {\begin{array}{*{20}c} {m_{1} + m_{2} + m_{3} } & 0 & { - (m_{2} + m_{3} )k_{1} \text{cos}(\omega_{3} )} & {m_{2} k_{2} \text{cos}(\omega_{4} )} & {m_{3} k_{\text{3}} \text{cos}(\omega_{5} )} \\ 0 & {m_{1} + m_{2} + m_{3} } & {(m_{2} + m_{3} )k_{1} \text{sin}(\omega_{3} )} & { - m_{2} k_{2} \text{sin}(\omega_{4} )} & { - m_{3} k_{3} \text{sin}(\omega_{5} )} \\ { - (m_{2} + m_{3} )k_{1} \text{cos}(\omega_{3} )} & {(m_{2} + m_{3} )k_{1} \text{sin}(\omega_{3} )} & {I_{1} + (m_{2} + m_{3} )k_{1}^{2} } & { - m_{2} k_{1} k_{2} \text{cos}(\omega_{3} - \omega_{4} )} & { - m_{3} k_{1} k_{3} \text{cos}(\omega_{3} - \omega_{5} )} \\ {m_{2} k_{2} \text{cos}(\omega_{4} )} & { - m_{2} k_{2} \text{sin}(\omega_{4} )} & { - m_{2} k_{1} k_{2} \text{cos}(\omega_{3} - \omega_{4} )} & {I_{2} + m_{2} k_{2}^{2} } & 0 \\ {m_{3} k_{3} \text{cos}(\omega_{5} )} & { - m_{3} k_{3} \text{sin}(\omega_{5} )} & { - m_{3} k_{1} k_{3} \text{cos}(\omega_{3} - \omega_{5} )} & 0 & {I_{3} + m_{3} k_{3}^{2} } \\ \end{array} } \right] \hfill \\ \end{aligned}$$

The equation for *B* is

$$B(W,\dot{W}) = \left[ {\begin{array}{*{20}c} 0 & 0 & {(m_{2} + m_{3} )k_{1} \dot{\omega }_{3} \text{sin}\omega_{3} } & { - m_{2} k_{2} \dot{\omega }_{4} \text{sin}\omega_{4} } & { - m_{3} k_{3} \dot{\omega }_{5} \text{sin}\omega_{5} } \\ 0 & 0 & {(m_{2} + m_{3} )k_{1} \dot{\omega }_{3} \text{cos}\omega_{3} } & { - m_{2} k_{2} \dot{\omega }_{4} \text{cos}\omega_{4} } & { - m_{3} k_{3} \dot{\omega }_{5} \text{cos}\omega_{5} } \\ 0 & 0 & 0 & {m_{2} k_{1} k_{2} \dot{\omega }_{4} \text{sin}(\omega_{4} - \omega_{3} )} & {m_{3} k_{1} k_{3} \dot{\omega }_{3} \text{sin}(\omega_{5} - \omega_{3} )} \\ 0 & 0 & {m_{2} k_{1} k_{2} \dot{\omega }_{3} \text{sin}(\omega_{3} - \omega_{4} )} & 0 & 0 \\ 0 & 0 & {m_{3} k_{1} k_{3} \dot{\omega }_{3} \text{sin}(\omega_{3} - \omega_{5} )} & 0 & 0 \\ \end{array} } \right]$$

The equation for the gravity vector is:

$$G(W) = \left[ {\begin{array}{*{20}c} 0 \\ { - m_{1} } \\ { - (m_{2} + m_{3} )k_{1} \text{sin}\omega_{3} } \\ { - m_{2} k_{2} \text{sin}\omega_{4} } \\ {m_{3} k_{3} \text{sin}\omega_{5} } \\ \end{array} } \right]$$

The coefficient matrix for the support force vector Γ is

$$\partial C/\partial W = \left[ {\begin{array}{*{20}c} 1 & 0 & 1 & 0 \\ 0 & 1 & 0 & 1 \\ { - k_{1} \text{cos}\omega_{3} } & {k_{1} \text{sin}\omega_{3} } & { - k_{1} \text{cos}\omega_{3} } & {k_{1} \text{sin}\omega_{3} } \\ {l_{2} \text{cos}\omega_{4} } & { - l_{2} \text{sin}\omega_{4} } & 0 & 0 \\ 0 & 0 & {l_{3} \text{cos}\omega_{5} } & { - l_{3} \text{sin}\omega_{5} } \\ \end{array} } \right]$$

The biped has three actuators:

$$U = \left[ {u_{12} ,u_{13} ,u_{23} } \right]$$

The matrix *E* is:

$$E = \left[ {\begin{array}{*{20}c} 0 & 0 & 0 \\ 0 & 0 & 0 \\ 1 & { - 1} & 0 \\ { - 1} & 0 & 1 \\ 0 & 1 & { - 1} \\ \end{array} } \right]$$

referring to Fig. 1; the box B.S. represents Eq. (12). The outputs of this box are the acceleration and velocities to be integrated with respect to time.

### Platform dynamics

The equations of the above system must be augmented with the dynamics of the platform. We assume the platform rotates about its center of gravity and is large enough and the actuators that implements its rotation are large. For simplicity, the equation of motion for the platform is taken to be a second-order system with one actuator.

14 $$I_{p} \ddot{\beta } + L_{p} \dot{\beta } + K_{p} \beta = v_{1} + v_{2}$$

The box labeled P.S. in Fig. 1 represents the scalar transfer function

$$\frac{Phi}{{U_{d} }} = \frac{1}{{(I_{p} s^{2} + L_{p} - ps + K_{p} )}}$$

The variable *v*_1_ represents the moment of the forces of constraint

$$v_{1} = R_{p} \varGamma$$

The variable *v*_2_ is the output of the controller: large gain proceeded by a PID controller. It is assumed that the output of the platform subsystem is the angle of rotation of the platform, and the input to the platform subsystem is the desired angle of rotation *β*, i.e.,

$$U_{d} = \beta$$

Standard control techniques can be applied to make the transfer function *Φ*/*v*_1_ small and the transfer function *Φ*/*U*_*d*_ close to identity. Our objective is to concentrate on the sensory and control aspects of the biped and, specifically, its postural stability in reaction to the platform disturbance. We further assume that

$$\varPhi = \beta$$

and would like to reduce the dynamics of the system to that of the biped alone and with the known kinematics of the platform motion. Contrast with a person on a skatingboard where the forces of contact and support are used to control the board.

### Platform kinematics plus biped dynamics

It is assumed that the platform actuator and moment of inertia are large enough such that influence of the forces of support on the platform dynamics is minimal and hence negligible. This assumption allows the motion analysis to be restricted to the kinematics of the platform motion of specific known trajectories.

The augmented system is characterized by six degrees of freedom:

$$W_{a} = \left[ {W,\beta } \right]$$

The coefficient matrices in Eq. (7) must in turn be augmented:

$$J_{a} ,B_{a} ,Gr_{a} ,\partial C/\partial W_{a} ,\;{\text{and}}\;E_{a}$$

The structure of each of these matrices is briefly described. The two-block diagonal matrix of inertia *J*_*a*_ is made up of *J* and *J*_*p*_. The matrix *B*_*a*_ is the two-block diagonal matrix of *B* and a zero. The column vector of gravity *Gr* is augmented by a zero, because we assume the platform is rotated about its center of gravity. The matrix *E*_*a*_ is extended by a sixth row of three zeros and one and by a fourth column of zeros except for the last element. This is to account for the platform acceleration as an input. The coefficient of Γ matrix will be augmented by a row of four zeros at the bottom. This merits some explanation. We are justified in taking this step since the dynamic equation of the platform does not depend on the forces of support. On the other hand when we differentiate the constraints with respect to *β* one gets a last row for *ӘC*_*a*_/*ӘW*_*a*_ that is not zero. The importance of this last matrix is in the calculation of the support forces. Because *J*_*a*_ is a block diagonal matrix with zeros in the last row and last column, the denominator of the computation of Γ will not depend on *β* as discussed earlier. The dependence of the forces of support on *β*, the angle of rotation, and its derivative and second derivative will be through the twice differentiation of the constraints with respect to time which leads to the direct dependence of the forces of support on the rotation angle, and its angular velocity and acceleration.

### Stability and control

Stability can be achieved by control of the stiffness of agonist–antagonist pairs at joints and across the segments [21]. The simultaneous co-activation of agonist–antagonist pairs of muscles at least by a pair at every joint achieves the same end. The co-activation produces sufficient position and velocity feedback to bring about stability in some vicinity of the equilibrium position. Lyapunov stability can be utilized to establish the domain of attraction, and the platform disturbances are modeled as a perturbation.

Let *K*_*d*_ be a diagonal 5 × 5 matrix whose diagonal elements are, respectively, the stiffnesses at the joints and across the joints. Similarly, let *K*_*p*_ be a diagonal matrix of joint and across joint viscosities. Let $\Delta \varTheta$ be defined as follows:

$$\Delta \varTheta = [\Delta \theta_{1} ,\Delta \theta_{2} ,\Delta \theta_{3} ]^{\prime}$$

The incremental work, ∆*W*, of the torques *U* = [*u*_12_, *u*_13_, *u*_23_] on the system is

$$\Delta W = - (\Delta \varTheta )E^{\prime}U^{\prime}$$

Thus, the input to the system will be

$$T = - E^{\prime}U^{\prime}$$

For the input torques, it is designed using the PD control as

$$U = K_{p} E\varTheta + K_{d} E\dot{\varTheta }$$

Therefore, the effect of co-activation of the three torque pairs can be represented as

$$T = - E^{{\prime }} K_{p} E\varTheta - E^{{\prime }} K_{d} E\dot{\varTheta } = - K_{kP} \varTheta - K_{kd} \dot{\varTheta }$$

where

$$K_{kp} = \left[ {\begin{array}{*{20}c} {k_{p1} + k_{p2} } & { - k_{p1} } & { - k_{p2} } \\ { - k_{p1} } & {k_{p1} + k_{p3} } & { - k_{p3} } \\ { - k_{p2} } & { - k_{p3} } & {k_{p2} + k_{p3} } \\ \end{array} } \right]$$

This matrix allows one to do quantitative analysis by selecting the joint and biarticular stiffnesses. The velocity feedback matrix *K*_*kd*_ is constructed by assuming as a diagonal matrix and is a fraction of the diagonal matrix *K*_*kp*_.

The above analytically shows that the co-activation of a pair of agonist–antagonist actuators results in negative angular position and angular velocity feedback. The stiffness and viscosity, i.e., the position and the velocity feedback gains, can be programmed. Thus, the system is activated by three quantities: stiffness and viscosity at the three joints among the three segments of the biped in Fig. 1. We consider the stability of the system about the vertical stance.

## Simulations and comparison

When the planar biped is on a rotating platform as in Fig. 1, the effect from the platform to the biped is the centrifugal force. In this case we only consider the balance in the biped plane and assume that the motion perpendicular to the biped plane is fixed. Thus, the system can be described as

15 $$\left[ {\begin{array}{*{20}c} {{\text{eq}}1} \\ {{\text{eq}}2} \\ {{\text{eq}}3} \\ {{\text{eq}}4} \\ {{\text{eq}}5} \\ \end{array} } \right] = J(W)_{5 \times 5} \left[ {\begin{array}{*{20}c} {\ddot{x}} \\ {\ddot{y}} \\ {\ddot{\theta }_{1} } \\ {\ddot{\theta }_{2} } \\ {\ddot{\theta }_{3} } \\ \end{array} } \right] - \left[ {\frac{\partial C}{\partial W}} \right]_{5 \times 4} \left[ {\begin{array}{*{20}c} {F_{2v} } \\ {G_{2v} } \\ {F_{3v} } \\ {G_{3v} } \\ \end{array} } \right] + B(W,\dot{W})\dot{W} - gG(W) - E\left[ {\begin{array}{*{20}c} {u_{12} } \\ {u_{13} } \\ {u_{23} } \\ \end{array} } \right] + \dot{\beta }^{2} {\text{Cen}} = 0$$

where Cen is the centrifugal force as

$${\text{Cen}} = \left[ {\begin{array}{*{20}c} {(m_{1} + m_{2} + m_{3} )r} \\ 0 \\ { - (m_{2} + m_{3} )(r + k_{1} \text{sin}\omega_{3} )k_{1} \text{cos}\omega_{3} } \\ { - m_{2} (r + k_{1} \text{sin}\omega_{4} )k_{2} \text{cos}\omega_{4} } \\ { - m_{3} (r + k_{1} \text{sin}\omega_{5} )k_{3} \text{cos}\omega_{5} } \\ \end{array} } \right]$$

where *r* is the distance from the center of mass of the biped to the platform rotation axis.

For two-feet support, the geometric constraints of the biped are

16 $$C = \left\{ {\begin{array}{*{20}l} {l_{2} \text{cos}\theta_{2} = l_{3} \text{cos}\theta_{3} } \\ {l_{2} \text{sin}\theta_{2} - l_{3} \text{sin}\theta_{3} = d} \\ \end{array} } \right.$$

From the rotating system, there is no translation resulting in $\ddot{x} = \ddot{y} = 0$. By taking double derivative of geometric constraint in Eq. [16], $\ddot{\theta }_{2} ,\ddot{\theta }_{3}$ will be obtained. Substitute these into Eq. (15), five equations with five unknowns ($\ddot{\theta }_{1} ,F_{2v} ,G_{2v} ,F_{3v} ,G_{3v}$) can be solved.

For single-foot support, (16) does not exist resulting in $\ddot{\theta }_{2} ,\ddot{\theta }_{3}$ as unknowns. At the same time, two of the four ground forces disappear when considering left or right foot supports the body. In this case, there are still five unknowns ($\ddot{\theta }_{1} ,\ddot{\theta }_{2} ,\ddot{\theta }_{3} ,F_{2v} ,G_{2v}$) (left foot support, for right foot support it is ($\ddot{\theta }_{1} ,\ddot{\theta }_{2} ,\ddot{\theta }_{3} ,F_{3v} ,G_{3v}$)) which can be solved from the five equations in (15).

The above analysis will be used in the following simulations covering both single-foot support and double-feet support balance with the platform rotation as disturbance. Parameter *I* refers to the moment of inertia in *Kgm* ^2^ about the center of gravity of the link. Parameter *m* refers to the mass of the link in *Kg*. Parameter *l* refers to the length of the link in m. Parameter *k* refers to the distance of the center of mass of the link from the lower joint. The system parameters are given as:

$$r = 3,l_{2} = 1,l_{3} = 1,k_{1} = 0.28,k_{2} = 0.46,k_{3} = 0.46,I_{1} = 3.25,I_{2} = 1.4,I_{3} = 1.4,m_{1} = 50,m_{2} = 12,m_{3} = 12,g = 10.$$

### **Case 1**

**Fig. 3:**
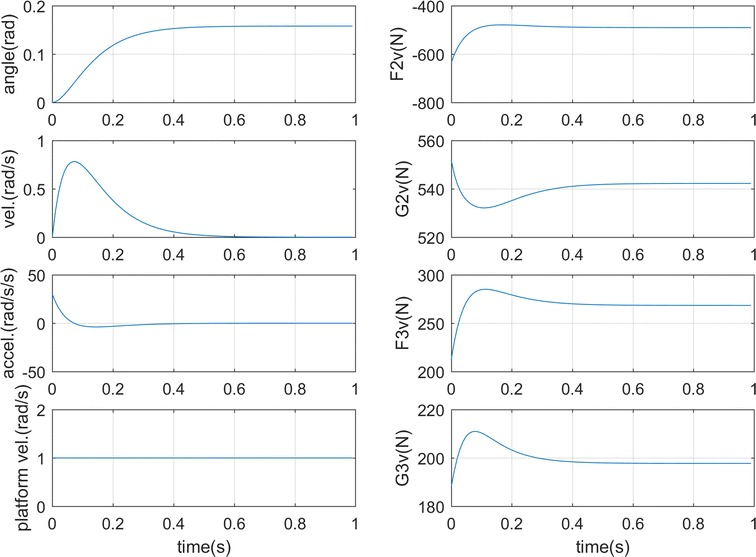
Simulation results for case 1. **a** Torso motion. **b** Ground forces

Two feet are on the platform and not moving, while the platform has a fixed angular velocity as 1 rad/s. The torso is the main moving part with *u*_12_ = −400 * *θ*_1_ − 40 $\dot{\theta }_{1}$, *u*_13_ = −400 * *θ*_1_ − 40 $\dot{\theta }_{1}$, *u*_23_ = 0 as the input using the negative position and velocity feedback [22]. The initial position is *θ*_1_ = 0, *θ*_2_ = 3π/4, *θ*_3_ = 5π/4. The simulation results show that the torso comes to the equilibrium position within 0.6 s as in Fig. 3a and the ground forces on the two feet also stabilize fast as shown in Fig. 3b. The ground forces [23] on the right foot increase to the equilibrium point, while those on the left foot decrease due to the double-feet support position and the centrifugal force of the platform pointing from left foot to the right. The simulation results conform to the human hip control strategies in disturbed standing [24, 25] by moving the center of mass anteriorly as seen from the θ_1_ value in Fig. 3a. A bigger right foot vertical force G2v than the left foot G3v also proves that the center of mass moves to the right. This is also in line with the analysis in [21] that the effect of a horizontal disturbance can be reduced when the torso bends to be parallel to the horizontal plane.

### **Case 2**

**Fig. 4:**
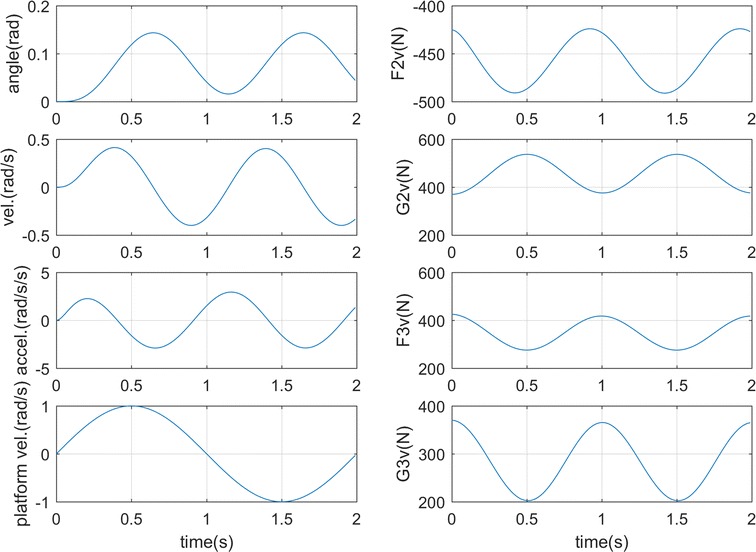
Simulation results for case 2. **a** Torso motion. **b** Ground forces

In this case, the platform angular velocity is set as sinusoidal function sin((2 * *π*/2) * *t*). The simulation result is shown in Fig. 4 which illustrates the stabilization with the input and initial position the same as in case 1. The torso can follow the platform disturbance after about 0.6 s, and the ground forces also balance with the process as in Fig. 4b. Since the centrifugal force constantly points from the left foot to the right foot, the hip adjusts the torso following the speed change of the platform to move the center of mass to the balanced point [24, 25] which is in the anterior side as seen from the sinusoidal wave of *θ*_1_ upon zero line in Fig. 3a. This can be also seen from the bigger right foot vertical force G2v varying between 380 and 540 N, and the combined horizontal force F2v and F3v keeps negative in the process due to the positive centrifugal force direction.

### **Case 3**

**Fig. 5:**
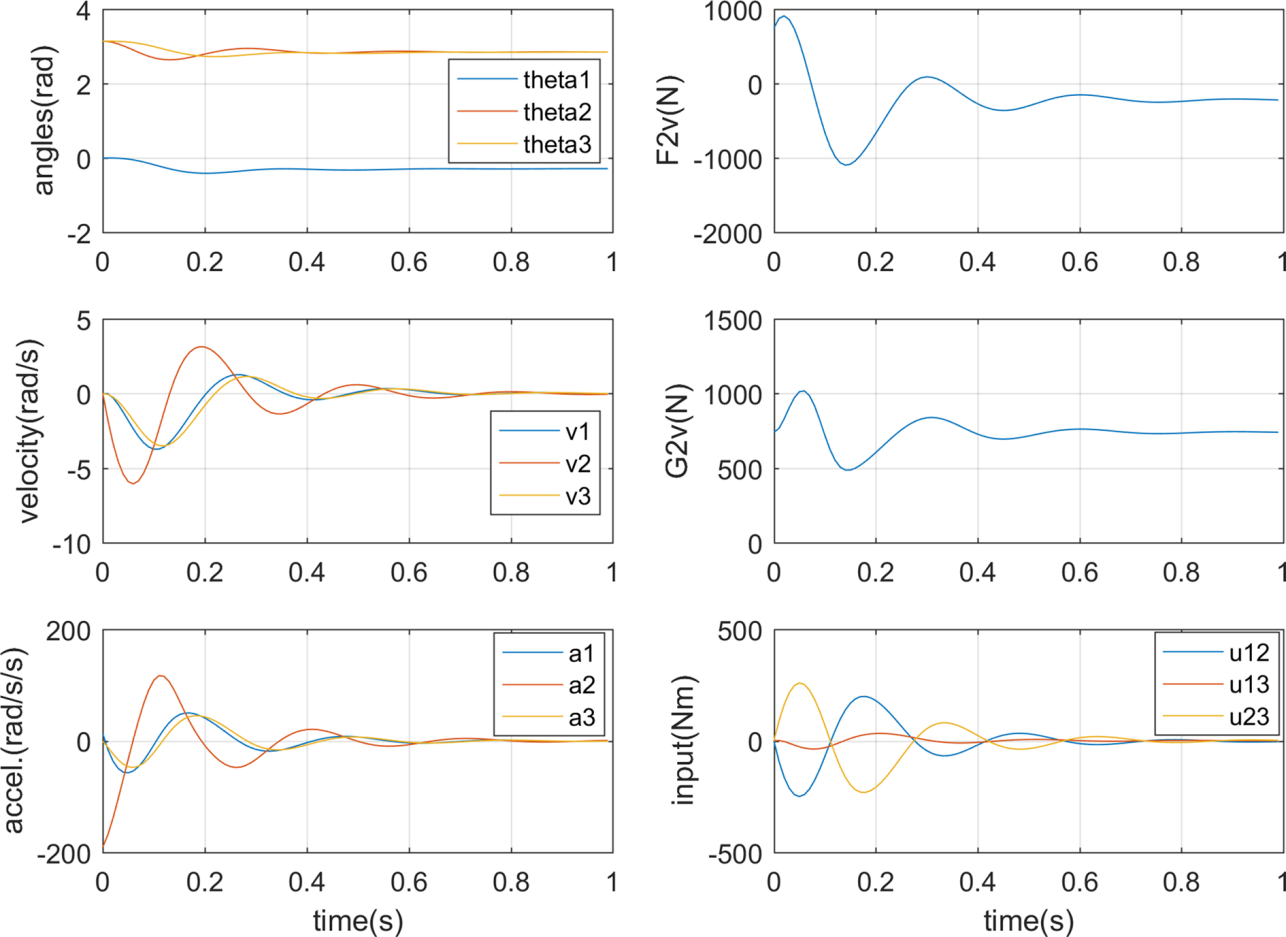
Simulation results for case 3. **a** Torso motion. **b** Ground forces and input torques

In this case only the right foot stands on the rotational platform to support the body, while the platform has a fixed angular velocity as 1 rad/s. The right leg is left free, and the body starts from the position *θ*_1_ = 0, *θ*_2_ = π, *θ*_3_ = π. The negative position and velocity feedback are chosen as *K* = [300, 300, 200] and *L* = [50, 50, 50]. The system stabilizes in about 0.8 s as shown in Fig. 5. The ground forces on the right foot also balance along the process, while the input torques for the three joints are illustrated in Fig. 5b. The horizontal platform disturbance is fully balanced by the horizontal ground force F2v, while the vertical ground force G2v mainly supports the body weight after the balance. The joint angles show that the torso moves the negative side (*θ*_1_ < 0) and both the legs move to the right (*θ*_2_, *θ*_3_ < *π*) of the vertical line. This conforms to the human ankle and hip strategy [26, 27] in disturbance standing in sagittal plane in which a double-segment inverted pendulum control strategy is used for the balance. When the body is balanced with the constant centrifugal force, the center of mass is moved to the opposite side of the centrifugal force direction as there is only one leg support and this is different to case 1 with two-foot support.

### **Case 4**

**Fig. 6:**
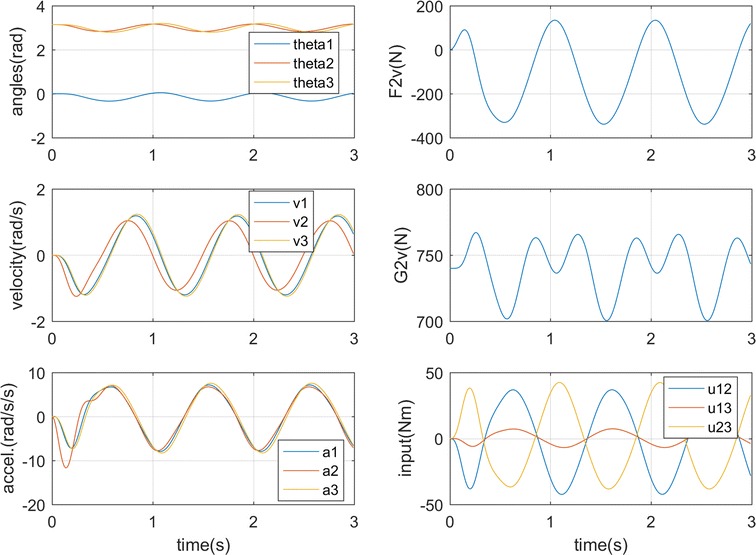
Simulation results for case 4. **a** Torso motion. **b** Ground forces and input torques

Following case 3, this case changes the platform angular velocity to sin((2 * *π*/2) * *t*). The simulation result is shown in Fig. 6 which illustrates the stabilization process in which the body follows the platform disturbance in less than 1 s. Similar to case 3, the horizontal ground force F2v resists the platform rotation force, while the vertical ground force G2v supports the body weight. It can be seen from the vertical ground force change that between two big peak changes a small peak exists due to the free left leg balancing. Input torques between − 22 and 18 N m are needed in the whole process. The results show similar strategy in the body balance with case 3 but with a sinusoidal process due to the sinusoidal balance. The body balances the center of mass between the left side of the vertical line and the right vertical position as seen from the joint angles in Fig. 6a.

## Discussion and conclusions

The work presented here explores the dynamics of separable coupled rigid body systems, a special class of constrained rigid body systems. Specifically, the dynamics of the two-coupled separable systems is formulated, and two examples, a biped model under rotational platform disturbances and a simple skateboard model, are analyzed for illustration and computationally simulated. The results of the simulations adequately validate the proposed theoretical models. The inertia forces, which resulted from the moving platform, can be decoupled and integrated into the body system as a disturbance, and the balance and stability of the body can be guaranteed with the stiffness and viscosity feedback control.

The main contribution of this work is the novel extension to the known dynamics of constrained rigid bodies. The modular, versatile and systematic formulation presented here is computationally efficient and has many applications in the studies of the human neuro-musculoskeletal system, robotic systems and humanoids [28], as well as clinical and sports biomechanics applications. Examples include clinical settings of patients interfacing with a rotating medical platform or machine, as well as the dynamics, stability and control of athletes on surf or diving boards. Further potential applications include quantitative studies on the balance and postural strategies as related to balance and vestibular dysfunction and deficits. Simulation and animation studies of human motion in interface with stationary or moving bases of support could also benefit from the work presented here. Examples include some of today’s extreme sports, such as skateboarding and surfboarding, where balancing and control are critical for safety.

The current simulation used a simplified three-rigid body link model, where only hip joint control strategies were included. Since the ankle strategy is typically adopted in human balance control modeling [21], a more complex musculoskeletal model that also includes the knee and ankle joints would allow for better biofidelic representation of human motion. Future work should also study the effect of the inertial forces of the moving base on the control strategies, as these forces are integral to physiological movement and human interaction with the environment. The motion of the moving base in this work was assumed as a simple rotation with constant velocity. Future work should consider more complex motions of the base, including translation, and a variety of speeds in order to simulate various real-life scenarios such as the interaction with an accelerating or turning vehicle. Future work would also benefit by including experiments with human participants for validating the simulation results and to extract more realistic motion control strategies.
